# Seasonal variations in gut microbiota of semiprovisioned rhesus macaques (*Macaca mulatta*) living in a limestone forest of Guangxi, China

**DOI:** 10.3389/fmicb.2022.951507

**Published:** 2022-09-20

**Authors:** Hongying Liu, Ting Chen, Yuhui Li, Jingjin Zheng, Zheng Liu, Youbang Li, Zhonghao Huang

**Affiliations:** ^1^Key Laboratory of Ecology of Rare and Endangered Species and Environmental Protection (Guangxi Normal University), Ministry of Education, Guilin, China; ^2^Guangxi Key Laboratory of Rare and Endangered Animal Ecology, Guangxi Normal University, Guilin, China; ^3^College of Life Sciences, Guangxi Normal University, Guilin, China

**Keywords:** gut microbiota, seasonal variations, community assembly, rhesus macaque, limestone forest

## Abstract

Assessment of gut microbiota, used to explore ecological adaptation strategies and evolutionary potential of species, provides a new viewpoint to the conservation and management of endangered animals. In this research, the gut microbiota of a group of semiprovisioned rhesus macaques (*Macaca mulatta*) living in a limestone forest exhibiting seasonal changes in plant items were studied to investigate the adaptation strategies of these macaques to this specific habitat. The findings revealed significant seasonal changes in the diversity and composition of the rhesus macaques’ gut microbiota, which were higher in the rainy season than in the dry season. In the rainy season, Bacteroidetes (31.83 ± 16.14% vs. 19.91 ± 18.20%) were significantly increased and *Prevotella* (23.70 ± 15.33% vs. 15.40 ± 16.10%), *UCG-002* (4.48 ± 3.16% vs. 2.18 ± 2.01%), and *UCG-005* (4.22 ± 2.90% vs. 2.03 ± 1.82%) were more enriched at the genus level. In the dry season, Firmicutes significantly increased (71.84 ± 19.28% vs. 60.91 ± 16.77%), and *Clostridium_sensu_stricto_1* (8.45 ± 9.72% vs. 4.76 ± 6.64%), *Enterococcus* (10.17 ± 13.47% vs. 0.69 ± 2.36%), and *Sarcina* (4.72 ± 7.66% vs. 2.45 ± 4.71%) were more enriched at the genus level. These differences in gut microbiota may be due to seasonal variations in plant items in these habitats alongside changes in the provisioned foods from tourists. Additionally, deterministic processes predominate the assembly of the macaque’s gut microbiota community. This indicates that the animal’s high reliance on natural plants and provisioned foods increased the impact of deterministic processes. This study concludes that a balance between provisioned foods and natural plants might be vital in shaping the gut microbiota in the macaques. Furthermore, the dynamic adjustment in gut microbiota might be a physiological mechanism for the macaques in response to the seasonal variations in the ecological factors and food provision.

## Introduction

The gut microbiota of animals is very diverse and plastic, forming a complex microbial community that interacts with the host and is mutually beneficial (Ley et al., 2006, 2008; Lozupone et al., 2012). Further research has shown that the diversity and composition of the gut microbiota affect the host’s health (Marchesi et al., 2016), digestion (Zhu et al., 2011; Flint et al., 2012), and behavior (Hsiao et al., 2013), and that it may even be considered in other essential organs (O’Hara and Shanahan, 2006). Many factors affect the composition and diversity of the gut microbiota, including diet (Filippo et al., 2010; David et al., 2014; Merra et al., 2021; Wang et al., 2022), host characteristics (Goodrich et al., 2014; Li et al., 2021), social contacts (Sarkar et al., 2020), and environment (Li et al., 2019; Wu et al., 2022). The diet is the most significant factor and even outweighs host genetics (Hale et al., 2018). Specifically, the gut microbiome regulates their vital metabolic functions to adapt to diet changes (David et al., 2014), facilitating animal species’ evolution (Ley et al., 2008; Moeller and Sanders, 2020). From an evolutionary viewpoint, new dietary changes are necessary to shape new species’ evolution (Ley et al., 2008). Food variations commonly entail changes in selection pressure, consequently resulting in alterations in the microbial community structure that reflects the dynamic nature of the bacteria (Muegge et al., 2011; David et al., 2014; Merra et al., 2021). Wild animals’ gut microbiota appears to fluctuate regularly due to seasonal variations in food sources (Amato et al., 2015; Orkin et al., 2019; Baniel et al., 2021). Usually, seasonal variations in plant items, primarily due to a shortage of fruits and young leaves, alter the dietary choices and foraging strategies of wild animals (Zhou et al., 2009; Huang et al., 2015), consequently resulting in structural changes and functional adjustments in the gut microbiota (Hicks et al., 2018; Orkin et al., 2019). For instance, Tibetan macaques (*Macaca thibetana*) respond to the lack of fruits by relying more on high-cellulose foods such as mature leaves and roots, increasing the *Succinivibrio* that aid in digesting cellulose in the foods (Sun et al., 2016). Therefore, seasonal variations in gut microbiota are considered an adaptive strategy in response to environmental changes (Lozupone et al., 2012; Baniel et al., 2021) as reported in other non-human primates like black howler monkeys (*Alouatta pigra*) (Amato et al., 2015), geladas (*Theropithecus gelada*) (Baniel et al., 2021), and white-faced capuchins (*Cebus imitator*) (Orkin et al., 2019).

Wildlife tourism has potentially adverse impacts on wild animals, partially due to the proximity to humans or receiving provisioned food, potentially leading to reduced fitness or imbalance of the gut microbiota (Clayton et al., 2016; Dallas and Warne, 2022). Usually, different food compositions considerably affect the structural features of the gut microbiota in both wild and captive animals (Clayton et al., 2016; Hale et al., 2018). For instance, increased gut microbiota diversity occurs in the wild red-shanked douc langur (*Pygathrix nemaeus*) than in the captive ones, probably due to their increased digestive capability to adapt to higher food diversity and dietary fiber consumption (Clayton et al., 2016). Therefore, exploring the impacts of food provisioning on the gut microbiota in protected animals is advantageous to understanding their digestive strategies and health, which could be included in subsequent conservation efforts.

Community assemblies are vital in microbial ecology as they shape and maintain microbial diversity (Zhou and Ning, 2017; Li et al., 2022). Presently, theories explaining the microbial community assembly include niche and neutral theories (Dini-Andreote et al., 2015; Mo et al., 2018). According to niche theory, the community assembly is a deterministic process influenced by ecological factors (Gilbert et al., 2012; Li et al., 2019). The neutral theory states that stochastic processes such as birth, death, migration, and ecological drift determine the community assembly (Bell, 2001; Sloan et al., 2006; Zhou and Ning, 2017). However, the combination of these two conflicting theories have explained better the factors influencing the community assembly (Dini-Andreote et al., 2015; Ning et al., 2020). The neutral community model (NCM) quantifies neutral processes that are complex to observe directly but affect microbial community assemblies, explaining the comparative significance of the deterministic and stochastic processes in diverse microbial communities (Bell, 2001; Sloan et al., 2006, 2007). It has been successfully applied to several microbial communities (Sloan et al., 2007; Mo et al., 2018). Research shows that increasing ambient temperatures in grassland microbial communities improve selection pressure, leading to the superiority of adaptive microorganisms (Ning et al., 2020). Moreover, the disparity in wild and captive white-lipped deer (*Cervus albirostris*) shows that the selective effect of feeding increases the relative importance of deterministic processes in the gut microbiota community assembly in captive animals (Li et al., 2022).

The digestive tract of rhesus macaques (*M. mulatta*) is characteristically frugivorous (Chivers and Hladik, 1980). Nevertheless, many studies have shown a high proportion of leaves in the macaques’ diets (Wang et al., 1994; Tang et al., 2011). The macaques live in limestone forests mainly feed on leaves. They are therefore susceptible to seasonal environmental variation, indicating a significantly higher intake of fruits and young leaves during the rainy season while they feed more on mature leaves, petioles, and bark during the dry season (Wang et al., 1994; Tang et al., 2011). In response to food availability changes, the gut microbiota of these macaques in the limestone forest might be similar to those of typical folivores (Chen et al., 2020). More so, the macaques received provisioned food, which varies with the number of tourists (Chen et al., 2019), potentially altering their gut microbiota. Limited research has focused on the gut microbiota of semiprovisioned rhesus macaques in limestone habitats (Chen et al., 2019; Li et al., 2021). Nevertheless, few studies have demonstrated the variations in the gut microbiota of these macaques in response to tourism and other ecological factors; hence, the adaptation strategies of the rhesus macaques in limestone habitats are unclear.

We studied the gut microbiota of the semiprovisioned rhesus macaques in a limestone forest using 16S rRNA high-throughput sequencing. We initially described the composition and diversity of the gut microbiota and then evaluated their seasonal changes. Next, the community assembly process of the gut microbiota was assessed using the NCM (Sloan et al., 2006). Lastly, these variations in the microbiota of the macaques were discussed by testing the following predictions:

(1) The complex diet improves gut microbiota diversity (Hale et al., 2018; Dallas and Warne, 2022). The blossoming growth of young leaves and fruits during the rainy season and the uncertainty of provisioning increased food diversity (Wang et al., 1994; Chen et al., 2019). We predicted that the gut microbiota diversity of the macaques in the rainy season is higher than that in the dry season.
(2) Seasonal variations in gut microbiota respond to changes in dietary composition (Orkin et al., 2019; Sun et al., 2020; Baniel et al., 2021). We predicted that the relative abundance of fat-digesting bacteria is increase in the gut of these macaques during the rainy season and cellulose-digesting bacteria during the dry season.
(3) Captive animals are subjected to environmental filtering imposed by feeding (Li et al., 2022). We predicted that provisioning foods increase the relative importance of deterministic processes in the gut microbiota assembly of the macaques.

## Materials and methods

### Study site and subjects

Guangxi Longhu Mountain Nature Reserve (hereafter “Longhu Mountain”) (22°56′–23°00′ N, 107°27′–107°41′ E), located in the southwest of Guangxi, China, is characterized by “karst” landform with a mean altitude of 300–500 m, covered by a subtropical mountain seasonal rainforest (Guangxi Forest Inventory and Planning Institute, 2005). The reserve belongs to the tropical monsoon climate region with an annual temperature of 21.8°C and mean annual rainfall of 1,500 mm, mainly occurring in summer and autumn (Guangxi Forest Inventory and Planning Institute, 2005). Based on the monthly rainfall, April to October is classified as the rainy season (mean monthly rainfall is 188.3 mm) and November to March as the dry season (mean monthly rainfall is 51.6 mm) (Longan County Meteorological Bureau, 2019).

The study subjects were a group of rhesus macaque in the Longhu Mountain. The dominant plants in this region include *Raderomachera sinica*, *Zeniainsignis*, *Ouoxylum indicum*, *Bombax malabarica*, *Choerospondias axillaris*, *Burreticdendron hsienmu*, *Carcimia paucinervis*, *Cephalolmappa sinensis*, and *Cinnamonum carcarea*, which are essential food resources for these macaques (Wang et al., 1994). The macaques consume 103 plant species annually, including 23 species regularly consumed (Wang et al., 1994). Furthermore, these macaques principally use leaves, fruits, and flowers, with seasonal variations in consumption, and they consume more fruits and young leaves in the rainy season and more mature leaves in the dry season (Wang et al., 1994). These macaques also receive cooked corn from the staff and fat-rich foods such as biscuits, bread, and drinks from tourists. Additionally, provisioned foods are usually closely associated with tourism prosperity, and peak tourism mainly occurs during the rainy months (General Office of the State Council of the People’s Republic of China, 2018; Chen et al., 2019).

### Sample collection and preservation

A total of 154 fecal samples were obtained from October 2018 to September 2019 using sterile tools such as gloves, bamboo sticks, and collection tubes (Supplementary Table 1). Only the middle portion of the feces that did not touch the ground was collected by bamboo sticks and then placed into sterile collection tubes. After recording the collected samples, they were immediately put into dry ice and then transferred to an ultralow-temperature refrigerator at –80°C for further assessment.

### DNA extraction, PCR amplification, and sequencing

We used E.Z.N.A.^®^ Soil DNA Kit (Omega Bio-Tek) to extract bacterial DNA from the fecal samples. The concentration and purity of the extracted DNA were determined using NanoDrop 2000 (Thermo Fisher Scientific), while DNA quality was checked using 1% agarose gel electrophoresis. The 16S rRNA gene V3–V4 region was amplified using bacterial primers (338F: 5′-ACTCCTACGGGAGGCAGCAG-3′ and 806R: 5′-GGACTACHVGGGTWTCTAAT-3′) (Mori et al., 2014) in a polymerase chain reaction (PCR) system (GeneAmp 9700) consisting of 5× FastPfu Buffer (4 μl), 2.5 mM dNTPs (2 μl), bovine serum albumin (0.2 μl), FastPfu polymerase (0.4 μl), template DNA (10 ng), and 0.8 μl of each primer (5 μM). The PCR products were eluted from 2% agarose gels, purified by AxyPrep DNA Gel Extraction Kit (Axygen Biosciences), quantified, and homogenized using Quantus Fluorometer (Promega) based on sequencing requirements. Lastly, the purified PCR fragments were sequenced using the Illumina Miseq PE300 platform (Illumina), conducted by Majorbio Cloud Platform^1^.

### Bioinformatics and statistical analysis

Trimmomatic was used to screen the raw FASTQ files and FLASH (v. 1.2.11)^2^ software was used to splice it. Firstly, we truncated the reads at a site with a mean quality score of <20 over a 50 base-pair (bp) window, removing reads less than 50 bp. We precisely matched the primers’ barcodes, allowing a mismatch of up to two nucleotides, discarding improper sequences, and merging overlapping sequences greater than 10 bp based on overlapping sequences. Operational taxonomic units (OTUs) clustering was performed on non-repeating sequences with 97% similarity by UPARSE (v.7.1)^3^ (Edgar, 2013), and UCHIME was employed to remove chimeras (Edgar et al., 2011). The RDP classifier algorithm (v.2.13)^4^ was used to classify 16S rRNA gene sequences based on the SILVA database (release 138)^5^ with a confidence threshold of 80%.

Rank-abundance curves, which show the richness and uniformity of the sequenced samples at the OTU level, were drawn using R statistical software (v. 3.3.1). The sizable horizontal range of the curve shows high species richness, whereas the smooth curve indicates even distribution. Rarefaction curves were plotted for all samples to test if the data is sufficient for sequencing, as shown by detecting most of the microbial information in the sample. Relative abundances of bacterial taxa were expressed as mean ± standard deviation, and a histogram showing community composition was derived. Alpha diversity analyses of the samples based on OTUs were computed using Mothur software (v.1.30.2),^6^ including two community diversity indices (Shannon, in*vs*impson), two community richness indices (ACE: Abundance-based coverage, Chao), and an index showing sequencing depth (Good’s coverage). To improve the linearity, the Shannon, in*vs*impson, ACE, and Chao values were transformed using log_10_ (x) (Warton and Hui, 2011). The Kruskal–Wallis sum-rank test was used to test the alpha diversity differences, and seasonal differences were visualized by R statistical software. Principal coordinate analysis (PCoA) at the OTU level was performed on all samples based on Bray–Curtis distance matrices by QIIME (v.1.9.1).^7^ Adonis analysis was used further to assess the diversity differences between the two seasons. The relative abundances of the gut microbiota community at the phylum and genus level between the two groups were evaluated using the Wilcoxon rank-sum test on SPSS 23.0, with significance of *p* < 0.05 and the *p*-value revised by the false discovery rate, expressed as the corrected *p*-value. The abovementioned data were operated on the Majorbio Cloud platform^1^. The NCM (Sloan et al., 2006) was constructed based on the OTUs, quantifying the relative importance of stochastic and deterministic processes in the gut microbial community assembly. The species dispersal (m) and the model fitting (0 < *R*^2^ < 1) of the whole microbial community were estimated, and when the neutral effect (i.e., stochastic processes) was *R*^2^ < 1, a non-neutral effect (i.e., deterministic processes) was used to explain the remaining part (1−*R*^2^). The confidence value of the fitting statistic was 95%.

## Results

### Sequencing quality assessment

**FIGURE 1:**
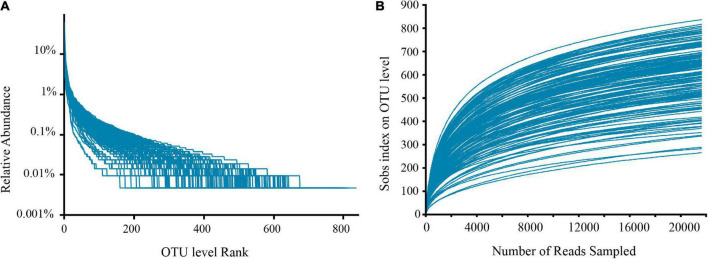
Rank-abundance curves **(A)** and rarefaction curves **(B)** show sufficient sequencing depth.

Filtering yielded 7,864,632 optimized sequences, and the original OTU table created following OTU cluster analysis had 5,906,920 sequences, corresponding to 38,356.60 ± 7,186.25 sample sequences per sample. Additionally, 1,708 OTUs were obtained for subsequent analysis. Rank-abundance (Figure 1A) and rarefaction curves (Figure 1B) were established basing on these OTUs, which showed that the sequencing included almost all species numbers observed in the fecal samples. Furthermore, good’s coverage estimators for all samples varied from 99.12 to 99.61%, signifying that the amount of sequencing data in this research was adequate, including most of the sample microbial information.

### Composition of gut microbiota in semiprovisioned rhesus macaques

**FIGURE 2:**
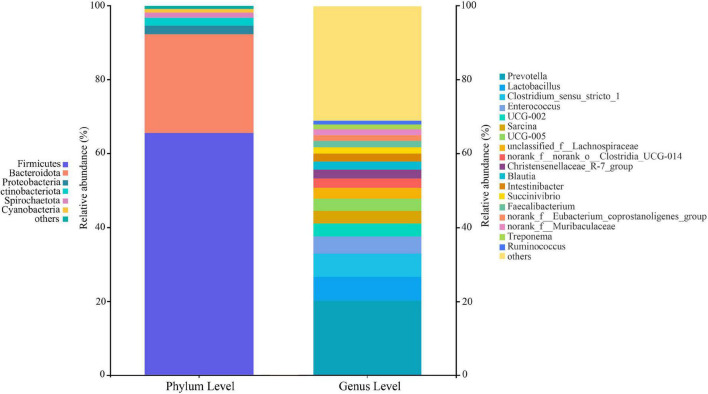
Composition of the gut microbiota at the phylum and genus levels. All taxa with a relative abundance of less than 1% were classified as “others”.

At the phylum level, 22 classified bacterial phyla were obtained from whole samples. The top four leading phylum include Firmicutes (65.52 ± 18.61%), Bacteroides (26.80 ± 17.98%), Proteobacteria (2.32 ± 4.02%), and Actinobacteria (2.13 ± 3.01%). At the genus level, 386 bacterial groups were obtained, and the dominant genera were *Prevotella* (20.20 ± 16.11%), followed by the *Clostridium_sensu_stricto_1* (6.43 ± 10.78%), *Lactobacillus* (6.31 ± 8.25%), and *Enterococcus* (4.69 ± 10.25%) (Figure 2). The proportions and relative abundances of other taxonomic groups at phylum and genus levels are listed in Supplementary Tables 2–5.

### Differences in gut microbiota diversity between rainy and dry seasons

**FIGURE 3:**
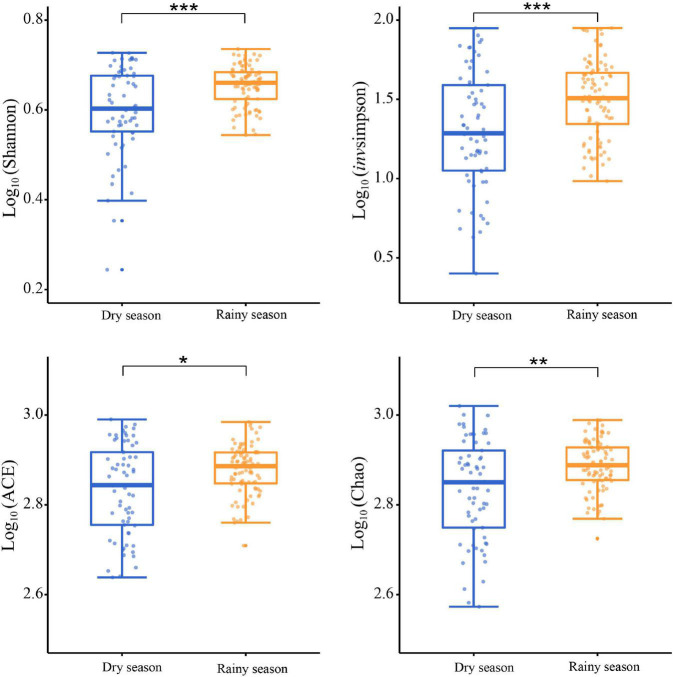
Comparison of the alpha diversity in gut microbiota between the dry and rainy seasons. Asterisks indicated a significant difference, with “*” for *p* < 0.05, “**” for *p* < 0.01, and “***” for *p* < 0.001.

**FIGURE 4:**
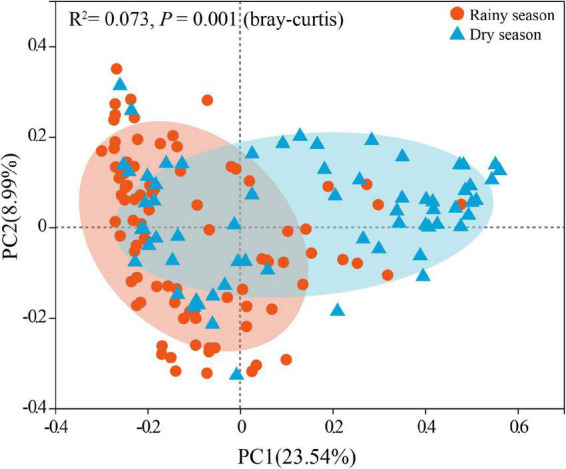
Seasonal comparison of gut microbiota beta diversity based on OTUs (Tested by Adonis).

After alpha diversity analysis, the Shannon index (4.34 ± 0.68), in*vs*impson index (32.88 ± 21.36), ACE (734.96 ± 127.74), Chao (746.38 ± 139.35) in the gut microbiota of all samples were obtained. Furthermore, alpha diversity exhibited noticeable seasonal variations. Particularly, the Shannon (χ^2^ = 11.463, df = 1, *p* < 0.001), invsimpson (χ^2^ = 11.290, df = 1, *p* < 0.001), ACE (χ^2^ = 6.016, df = 1, *p* = 0.014), and Chao (χ^2^ = 7.279, df = 1, *p* = 0.007) indices from the rainy season samples were markedly higher than those in the dry season, signifying a marked increase in the diversity, evenness, and richness of gut microbiota in rainy season (Figure 3). According to the Bray–Curtis distance (*R*^2^ = 0.073, *p* = 0.001), the beta diversity of the gut microbiota revealed significant seasonal separation (Figure 4).

### Differences in the gut microbiota community composition between rainy and dry seasons

**FIGURE 5:**
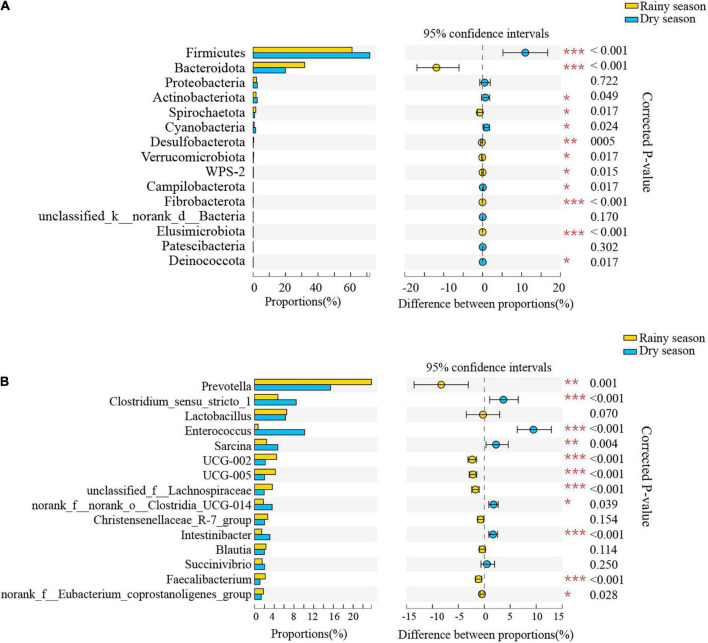
The composition difference analysis in the gut microbiota community at the phylum **(A)** and genus **(B)** levels. Only the top fifteen bacterial taxa are displayed. Asterisks indicated a significant difference, with “*” for *p* < 0.05, “**” for *p* < 0.01, and “***” for *p* < 0.001.

The results of the Wilcoxon rank-sum test showed significant differences in the relative abundance of bacteria from 12 phyla and 132 genera between the two seasons (Supplementary Tables 4, 5 and Figure 5). At the phylum level, Bacteroidetes, Spirochaetes, and Desulfobacterota were significantly higher proportion in the rainy season, while Firmicutes, Cyanobacteria, and Campilobacterota were in higher proportions in the dry season. At the genus level, *Prevotella*, *UCG-002*, *UCG-005*, and unclassified_f__Lachnospiraceae were more common in the rainy season, whereas *Clostridium_sensu_stricto_1*, *Enterococcus*, *Sarcina*, and norank_f__norank_o__Clostridia_UCG-014 were more enriched during the dry season.

### Community assembly of gut microbiota

**FIGURE 6:**
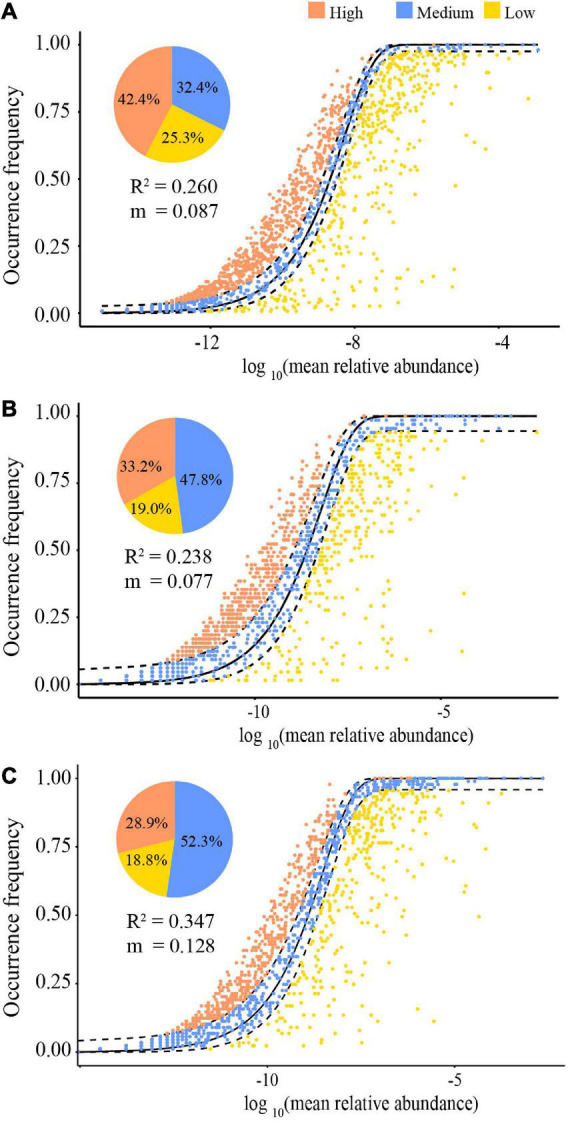
The quantitative results of stochastic processes in the community assembly of the gut microbiome in all samples **(A)**, dry season **(B)**, and the rainy season **(C)**, based on the NCM (the solid black lines represent the best fit to the NCM, and the dashed black lines represent 95% confidence intervals around the model prediction. OTUs that occur more or less frequently than predicted by the NCM are shown in different colors. “m” represents the migration rate, and “R^2^” represents the fitting to the model).

The NCM showed that stochastic processes might contribute to 26.0% (*R*^2^ = 0.260) (Figure 6A), 23.8% (*R*^2^ = 0.238) (Figure 6B), and 34.7% (*R*^2^ = 0.347) (Figure 6C) for all, dry and rainy season samples of the community assembly, respectively. The assembly of the gut microbiota communities in both seasons differed. Particularly, the contribution of stochastic processes in the rainy season was higher than that in the dry season (34.7 vs. 23.8%), and the species migration rate in the rainy season was higher than that in the dry season (*m* = 0.128 vs. *m* = 0.077). Furthermore, the NCM also computed the proportions of bacterial taxa in the confidence interval, and those of other taxa deviated from the confidence interval (Figure 6). During the dry season, 47.8% species and 52.3% during the rainy season were within the anticipated range, with the remaining species being outliers (Figure 6).

## Discussion

### Characteristics of the gut microbiota of semiprovisioned rhesus macaques

Firmicutes and Bacteroidetes dominate the gut microbiota in the rhesus macaques, as reported in other primates (Sun et al., 2016; Hale et al., 2019; Li et al., 2021), which are followed by Proteobacteria and Spirochaetes. Most Firmicutes bacteria species produce a range of digestive enzymes to digest ingested materials. Examples of such bacteria include *Lactobacillus* (Schoeler and Caesar, 2019) and *Clostridium_sensu_stricto_1* (Guo et al., 2020), probable supporting the digestion of the host. The bacteria in Bacteroidetes contribute to the degradation of carbohydrates and proteins in foods (Filippo et al., 2010; Flint et al., 2012). These two bacterial taxa were predominant in this study and might impact the digested and access to nutrients. Specifically, the macaques in limestone forests primarily obtain leaves as food (Wang et al., 1994), in which the decomposition of cellulose is inseparable from their gut microbiota. Proteobacteria, Actinobacteria, and Spirochaetes are the common taxa in the animal guts (Hicks et al., 2018; Li et al., 2019; Wang et al., 2022). Although some bacteria in Proteobacteria are associated with diseases (Marchesi et al., 2016; Orkin et al., 2019), we discovered that the *Succinivibrio*, which dominates, is committed to breaking down the polysaccharides and enhancing the degradation of food (Baniel et al., 2021). These bacteria taxa with lower relative abundances are also involved in maintaining the balance of the host’s gut microbiota (Tong et al., 2019).

Our findings revealed that the gut microbiota in the semiprovisioned macaques was maintained in high diversity, comparable to the results from previous research on the wild and provisioned macaques (Chen et al., 2019). Maintaining high gut microbiota diversity is essential for maintaining diverse digestive functions (Lozupone et al., 2012; Amato et al., 2013), signifying that dietary supplementation could not reduce the capacity of these macaques to ferment natural plants. Furthermore, early studies have discovered that replicating natural food helps maintain the diversity in the gut microbiota of captive animals (Martínez-Mota et al., 2020; Dallas and Warne, 2022). Nevertheless, overreliance on provisioned foods may increase the risk of obesity and steadily reduce the survival ability of wild animals (Clayton et al., 2016). Thus, balancing provision and natural feeding is necessary to maintain the ability of macaques to digest numerous food types, which is also the focus of animal rescue, protection, and management (Dallas and Warne, 2022).

### Seasonal changes in gut microbiota

We observed that the diversity of gut microbiota of the semiprovisioned rhesus macaques in the limestone forest was significantly higher in the rainy season than in the dry season, supporting prediction 1. The seasonal differences in plant resources brought on by phenology changes may cause this trend (Amato et al., 2015; Serra-Maluquer et al., 2021). Previous research has shown that limestone forests show significant seasonal changes in plant products, with fruits and young leaves usually appearing during the rainy season (Zhou et al., 2006; Huang et al., 2015). Assamese macaques (*Macaca assamensis*) (Huang et al., 2015), and François’ langur (*Trachypithecus francoisi*) (Zhou et al., 2006) living in the forests, typically use more fruits and young leaves during the rainy season. The macaques have reportedly undergone comparable dietary adjustments (Wang et al., 1994; Tang et al., 2011). Thus, this season’s comparatively abundant food resources can be connected to the increased gut microbiota diversity.

Tourism might also impact the differences in the gut microbiota. The macaques demonstrate higher gut microbiota diversity during the flourishing tourism season. Rainy seasons are typically busy tourism times as Chinese holidays predominantly overlap with the season, such as Sanyuesan in April, May Day, China’s National Day in October, and summer vacation in July and August (General Office of the State Council of the People’s Republic of China, 2018), which implies that the macaques get a more provisioned food during this season (Altmann and Muruth, 1988; Chen et al., 2019). While the semiprovisioned rhesus macaques depend on natural food resources, their diets typically include provisioned foods from the tourists, which could be linked to the variety in their diets; thus, providing a broad dietary niche that helps maintain high gut microbiota diversity (Amato et al., 2013; Chen et al., 2019; Martínez-Mota et al., 2020). Actually, the bacterial diversity in the rhesus macaques varies from other wild and captive groups, mainly due to their diet variations (Borbón-García et al., 2017; Hale et al., 2019).

Seasonal variations in the gut microbiota composition of the semiprovisioned rhesus macaques at both the phylum and genus levels were observed. The dominant bacterial taxa’s approximate change in relative abundance was consistent with prediction 2. The proportion of Firmicutes was significantly higher in the dry season than in the rainy season, and the common taxa in Firmicutes, such as Lachnospiraceae and Clostridiaceae, are necessary for cellulose decomposition in herbivorous animals (Ley et al., 2008; Wang et al., 2022). Notably, these bacteria can degrade numerous complex polysaccharides to produce short-chain fatty acids for easy absorption, provide energy to the host (Zhu et al., 2011; Hale et al., 2019). Increased Firmicutes in this study may be because these macaques gather more mature leaves, which are rich in cellulose, during the dry season to make up for the absence of young leaves and fruits (Wang et al., 1994). At the genus level, the proportions of *Clostridium_sensu_stricto_1*, *Enterococcus*, and *Sarcina* were significantly higher in the dry season. These bacteria are devoted to the degradation of indigestible polysaccharides, particularly cellulose (Zhu et al., 2011; Guo et al., 2020), which is consistent with the higher fiber diet of the macaques during the dry season. The selection pressure is changed by a high-fiber diet, and adding cellulose-digesting bacteria may help primates maintain their energy levels and enhance digestion (Hicks et al., 2018; Orkin et al., 2019; Baniel et al., 2021). Nevertheless, *Enterococcus* has often been found in some patients’ intestines with diarrhea and is considered unsafe (Marchesi et al., 2016). Therefore, the possible adverse effects of food supplementation in rhesus macaques must be further confirmed.

The proportion of Bacteroidetes was significantly higher in the rainy season than in the dry season, probably due to the increase in fatty, protein-rich food, such as peanuts and biscuits, by tourists. This comparatively high-fat diet enriches Bacteroidetes (Schoeler and Caesar, 2019; Sun et al., 2020) as this species can efficiently degrade carbohydrates and proteins in foods (Ley et al., 2006; Flint et al., 2012). Typically, caged primates fed a diet high in protein and low in fiber have more Bacteroides in their stomachs (Clayton et al., 2016; Borbón-García et al., 2017). Moreover, increased Bacteroidetes may be connected to increased availability of fruits, including vigorous growing fruits found in the limestone forest and peanuts fed by tourists during the rainy season (Wang et al., 1994), which are considered vital lipid sources in primates (Rothman et al., 2012). Similarly, ingestion of fruit by white-faced capuchins increases Bacteroidetes (Orkin et al., 2019). At the genus level, the enrichment of *Prevotella* primarily represents the increase in Bacteroidetes, which can divide substances such as xylan, pectin, and hemicelluloses (Flint et al., 2012), which are rich in young leaves and fruits (Filippis et al., 2016), justifying its dominance in the guts of semiprovisioned rhesus macaques owing to a considerably higher intake of young leaves and fruits during the rainy season. This shows that natural plants are still the staple foods, regardless of receiving the provisioned foods.

We also discovered that the cellulose-fermenting bacteria belonging to Firmicutes, such as *UCG-002*, *UCG-005*, and unclassified_f__Lachnospiraceae, is still high in relative abundance during the rainy season (Guo et al., 2020; Liao et al., 2021; Wang et al., 2022). This trend is consistent with their multifaceted diet with provisioned and natural foods. The digestion of ingested food and variations in the number of metabolic bacterial taxa typically cause the differences in the gut microbiota (Borbón-García et al., 2017). Thus, critical seasonal changes in gut microbiota can be a strategy in response to the seasonal variations in food resources of the macaques in limestone forests.

### Community assembly in gut microbiota

The relative importance of the deterministic processes in community assembly of all samples was 74.0%, signifying that it primarily influenced the clustering of gut microbiota in semiprovisioned rhesus macaques and that the assembly of gut microbiota was controlled by niche factors like environmental factors and interspecific interactions (Li et al., 2019; Wu et al., 2022). For instance, low-temperature filtration enhances deterministic processes to dominant in the gut the microbiota community assembly of wild pika (*Ochotona curzoniae*) at high altitudes (Li et al., 2019). Based on the seasonal variations in plant products and fluctuations in provisioning (Wang et al., 1994; Chen et al., 2019), it can be rationally speculated that diet alterations drive structural and functional adjustments in gut microbiota (David et al., 2014; Hale et al., 2018). Therefore, the increased contribution of the deterministic process is connected to the diet of semiprovisioned rhesus macaques. Because these macaques rely heavily on mature leaves during the dry season and primarily consume young leaves and fruits during the rainy season (Wang et al., 1994; Zhou et al., 2009), the influence of niche determinants on community formation is increased. Furthermore, the environmental filtering effect imposed by artificial feeding expands the contribution of deterministic processes (Li et al., 2022). This validates our prediction 3 about the gut microbiota community assembly in semiprovisioned rhesus macaques.

The fitting power of the model implies that deterministic factors are more able to explain the changes in gut microbial communities in the dry season, possibly due to comparatively stable food sources in the limestone habitats during the season, including the year-round supply of mature leaves and corn by staff (Wang et al., 1994; Chen et al., 2019). Like the dynamics of marine microbial communities that are impacted by two environmental factors: day length and temperature (Gilbert et al., 2012), food availability might be a significant environmental factor driving the gut microbiota community assembly (Li et al., 2022). Underfeeding and unstable aquaculture systems, zebrafish (*Danio rerio*) environmental selection pressure increases. Their gut microbiota community assembly tends to be dominated by deterministic processes (Burns et al., 2016). Numerous studies have also revealed that changes in the gut microbiota of wild animals are influenced by changes in food availability (Orkin et al., 2019; Baniel et al., 2021).

The diffusion of the gut microbiota of the macaques is less limited and is more likely to spread between individuals during the rainy season than in the dry season. Two possible explanations exist for this. First, according to Sloan et al. (2007) and Mo et al. (2018), numerous bacterial taxa in the community are more likely to experience an arbitrary dispersion across group members, which may be facilitated by the greater diversity and quantity of gut microbiota during the rainy season. Second, the distance between individuals may prevent bacterial taxa from migrating stochastically between individuals (Sarkar et al., 2020). The macaques living in limestone forests spend more time playing during the rainy season (Tang et al., 2011), and food supplementation significantly reduces the time spent foraging in these animals, with increased social and relaxation time (Altmann and Muruth, 1988). Therefore, comparatively intimate social contacts between group individuals increase the likelihood of gut microbiota migration during the rainy season, making the gut microbiota of individuals with close contacts more similar (Sarkar et al., 2020).

Other ecological factors did not impact the taxa found in the confidence interval (Sloan et al., 2007). Our findings showed that more taxa are in a stochastic state in the macaques’ gut microbiota community during the rainy season, which is also reflected by the fitting and species dispersal of the NCM. Microbial taxa differing from confidence intervals over three periods, also called “deviating taxa,” may be more vulnerable to the deterministic processes in the whole community, signifying potential ecological significance (Sloan et al., 2007). Particularly, more bacterial taxa in the dry season were beyond the confidence interval, which may be attributed to hosting the gut microbiota adaptation to the comparatively low-quality food resources in the dry season (Burns et al., 2016; Tong et al., 2019). The proportion of taxa lower the confidence interval was slightly higher in the rainy season, and these taxa were sustained despite being connected with potential pathogenic characteristics (Burns et al., 2016). The deviating taxa might interact with other taxa to increase the stability of the community structure in response to seasonal variations of the microbial community (Tong et al., 2019).

In summary, the significant seasonal changes in the diversity and composition in the gut microbiota of semiprovisioned rhesus macaques might be connected to the seasonal variations in plant items availability in the limestone forests and the changes in provisioned food from tourists. Furthermore, provisioning raises environmental selection pressure, with an increase in the relative importance of deterministic processes in the gut microbiota assembly of the macaques. Our results emphasize that diet is an essential factor impacting gut microbiota and that the flexible modulation in gut microbiota by the macaques might be an adaptive strategy in response to the seasonal variations in food availability. Additionally, we state that preserving natural foods in animal diets to maintain structural and functional diversity in the gut microbiota aids the primates in surviving environmental variations.

## Data availability statement

The datasets presented in this study can be found in online repositories. The names of the repository/repositories and accession number(s) can be found below: https://www.ncbi.nlm.nih.gov/, PRJNA863230.

## Ethics statement

The study was wholly non-invasive and did not involve any tissue collection. With the permission of Longhu Mountain (1450119M0030-0000986), we were allowed to enter the study site to collect samples. All fecal samples were collected after the animals had left to avoid stressing them.

## Author contributions

ZH and YL designed the study and revised the manuscript. HL analyzed the data and wrote the manuscript. TC, YuL, JZ, and ZL collected the samples. All authors read and approved the submitted manuscript.

## Footnotes

1
https://cloud.majorbio.com/

2
https://ccb.jhu.edu/software/FLASH/index.shtml

3
http://drive5.com/uparse

4
https://sourceforge.net/projects/rdp-classifier/

5
https://www.arb-silva.de/

6
https://www.mothur.org/wiki/Download_mothur

7
http://qiime.org/install/index.html
