# Supplementary material for: Seasonal variations in gut microbiota of semiprovisioned rhesus macaques (Macaca mulatta) living in a limestone forest of Guangxi, China
Source: Front Microbiol. 2022 Sep 20;13:951507. doi: 10.3389/fmicb.2022.951507 (PMC9530203; doi:10.3389/fmicb.2022.951507)
Supplement: Supplementary file 1 [file Table_1.DOCX]

Supplementary Table 1 Information on 154 fecal samples of rhesus macaques

| Month | Samples | Sex | | |  | Age | | |
| --- | --- | --- | --- | --- | --- | --- | --- | --- |
|  |  | Male | Female | Unknown |  | Adult | Immature | Unknown |
| Oct. 2018 | 17 | 2 | 2 | 13 |  | 15 | 2 | - |
| Nov. 2018 | 18 | 1 | - | 17 |  | 1 | - | 17 |
| Dec. 2018 | 16 | - | 4 | 12 |  | 16 | - | - |
| Jan. 2019 | 6 | - | - | 6 |  | 4 | 2 | - |
| Feb. 2019 | 13 | 4 | 3 | 6 |  | 3 | 10 | - |
| Mar. 2019 | 12 | 3 | 9 | - |  | 10 | 2 | - |
| Apr. 2019 | 11 | 6 | 5 | - |  | 7 | 4 | - |
| May. 2019 | 12 | 8 | 4 | - |  | 5 | 7 | - |
| Jun. 2019 | 12 | 3 | 9 | - |  | 10 | 2 |  |
| Jul. 2019 | 12 | 4 | 7 | 1 |  | 7 | 5 | - |
| Aug. 2019 | 13 | 5 | 7 | 1 |  | 10 | 3 | - |
| Sep. 2019 | 12 | 8 | 4 | - |  | 7 | 4 | 1 |
| Total | 154 | 44 | 54 | 56 |  | 95 | 41 | 18 |
